# Recent advances in understanding the biology of marginal zone lymphoma

**DOI:** 10.12688/f1000research.13826.1

**Published:** 2018-03-28

**Authors:** Francesco Bertoni, Davide Rossi, Emanuele Zucca

**Affiliations:** 1 Università della Svizzera italiana, Institute of Oncology Research, Bellinzona, Switzerland; 2 Oncology Institute of Southern Switzerland (IOSI), Ospedale San Giovanni, Bellinzona, Switzerland

**Keywords:** marginal zone lymphoma, genetics and biology of MZLs, extranodal MZL of MALT type, splenic MZL, nodal MZL

## Abstract

There are three different marginal zone lymphomas (MZLs): the extranodal MZL of mucosa-associated lymphoid tissue (MALT) type (MALT lymphoma), the splenic MZL, and the nodal MZL. The three MZLs share common lesions and deregulated pathways but also present specific alterations that can be used for their differential diagnosis. Although trisomies of chromosomes 3 and 18, deletions at 6q23, deregulation of nuclear factor kappa B, and chromatin remodeling genes are frequent events in all of them, the three MZLs differ in the presence of recurrent translocations, mutations affecting the NOTCH pathway, and the transcription factor Kruppel like factor 2 ( *KLF2)* or the receptor-type protein tyrosine phosphatase delta ( *PTPRD*). Since a better understanding of the molecular events underlying each subtype may have practical relevance, this review summarizes the most recent and main advances in our understanding of the genetics and biology of MZLs.

## Introduction

In the World Health Organization classification, there are three different marginal zone lymphoma (MZL) entities with specific diagnostic criteria, behavior, and therapeutic implications: the extranodal MZL of mucosa-associated lymphoid tissue (MALT) type (MALT lymphoma), the splenic MZL (SMZL), and the nodal MZL (NMZL) ^1^. MALT lymphoma is the commonest MZL type, accounting for 5 to 8% of all B-cell lymphomas ^2, 3^. Their differential diagnosis is not straightforward in the non-rare cases presenting with disseminated disease involving lymph nodes, spleen, peripheral blood, bone marrow, or other extranodal sites. A better understanding of the molecular events underlying each subtype may have practical relevance.

MZLs are believed to derive from B cells of the “marginal zone”, the external part of the secondary lymphoid follicles. The marginal zone is more evident in the lymphatic tissues continuously exposed to external antigens, such as the mesenteric lymph nodes, the MALT, and the spleen. Marginal zone B cells act as innate-like lymphocytes able to mount rapid antibody responses to both T cell–dependent and T cell–independent antigens, mostly the latter ^4^.

**Figure 1.**
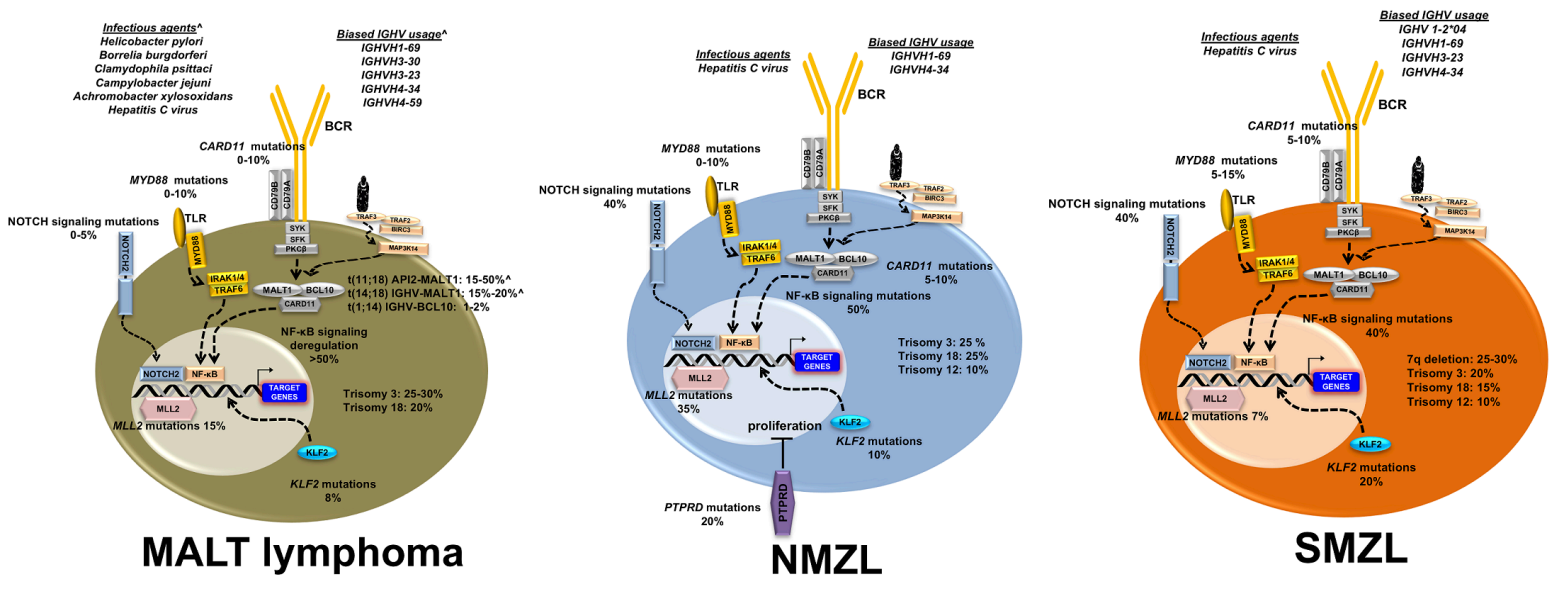
Summary of the main genetic and biologic features characterizing marginal zone lymphomas. ^Depending on the anatomical site. BCR, B-cell receptor; IGHV, immunoglobulin heavy variable; MALT, mucosa-associated lymphoid tissue; NF-κB, nuclear factor kappa B; NMZL, nodal marginal zone lymphoma; SMZL, splenic marginal zone lymphoma; TLR, Toll-like receptor.

The three MZLs clearly share common lesions and deregulated pathways, but they also present specific alterations that can be used for their differential diagnosis ^5– 24^ ( Figure 1). Trisomies of chromosomes 3 and 18 and deletions at 6q23 are frequent events in all MZLs, as well as somatic mutations of genes coding for proteins involved in chromatin remodeling ^5– 7, 25– 28^. The activation of the nuclear factor kappa B (NF-κB) pathway is also common to all three entities, generally via somatic mutations or deletions (or both) of *TNFAIP3* (A20) at 6q23 ^5– 7, 25– 27, 29– 33^. Mutations affecting the NOTCH pathway and the transcription factor *KLF2* are present in both SMZL and NMZL ^6, 7^. Whereas SMZL is specifically characterized by deletions of chromosome 7q, NMZL shows inactivation of *PTPRD* and a much higher prevalence of mutations affecting *KMT2D* (MLL2) ^6, 7, 32, 33^. Unlike the vast majority of other B-cell lymphomas, SMZL and NMZL do not present specific recurrent chromosomal translocations, while these are detected in MALT lymphomas, in which at least three of them activate the NF-κB pathway ^8– 14, 16, 34, 35^ ( Figure 1).

We will now highlight the most recent and main advances in our understanding of the genetics and biology of MZLs.

## NF-κB signaling

Active NF-κB signaling is necessary for the generation and maintenance of normal marginal zone B cells and this requires weak B-cell receptor (BCR) signaling (for example, started by auto-antigens and leading to canonical NF-κB pathway activation) or CD40 signaling, activating the non-canonical NF-κB pathway ^36, 37^. Following BCR engagement, Src family kinases phosphorylate the cytoplasmic ITAM portions of CD79A and CD79B ^38– 45^. The latter bind the tyrosine kinase SYK and start a signaling cascade that, via the Bruton’s tyrosine kinase (BTK), results in phosphorylation and activation of CARD11. CARD11, BCL10, and MALT1 form the CBM signaling complex linking BCR signaling to the canonical NF-κB pathway. Upon phosphorylation, CARD11 acquires an open conformation, allowing the recruitment of CARD11 to MALT1 and BCL10 into the CBM complex and activate the IKBKB kinase. IKBKB phosphorylates the IκBα inhibitor molecule, causing its proteasome-mediated degradation. Finally, the NF-κB complexes (mainly p50/RelA and p50/c-Rel dimers) can enter the nucleus and act as transcriptional factors. TNFAIP3 negatively regulates the whole pathway, adding and subtracting ubiquitin moieties to different NF-κB signaling pathways. Binding of CD40 activates the non-canonical NF-κB pathway. Following disruption of a negative regulatory complex comprising TRAF3/MAP3K14-TRAF2/BIRC3, the MAP3K14 kinase (also known as NIK) phosphorylates NFKB2 (p100), causing its proteasomal processing and the formation of p52-containing NF-κB dimers. In particular, BIRC3 (cIAP2), owing to its C-terminal RING domain, has ubiquitin ligase (E3) activity ^46^ and leads to BCL10 and MAP3K14 ubiquitination ^46^. Similarly, TRAF3 induces MAP3K14 degradation by recruiting it to the BIRC3 ubiquitin ligase complex. The p52 protein dimerizes with RelB to translocate into the nucleus, acting as a transcriptional factor.

In all of the MZLs, both canonical NF-κB signaling and non-canonical NF-κB signaling are deregulated by genetic events. The most frequent event is the inactivation, by deletions or mutations, of its negative regulator encoded by the *TNFAIP3* (A20) gene ^5, 6, 25– 27, 29– 31, 47^. Three other NF-κB signaling components— *MALT1*, *BCL10*, and *BIRC3*—are involved in the three most recurrent MALT lymphoma translocations: the t(11;18)(q21;q21), the t(14;18)(q32;q21), and the (1;14)(p22;q32) ^46, 48^. The t(11;18) translocation creates the BIRC3-MALT1 fusion protein, in which BIRC3 always lacks its RING domain; thus, BIRC3-MALT1 can bind BCL10 through the BIR domains of BIRC3 but no longer ubiquitinates BCL10 ^46^. BIRC3-MALT1 can also mediate the proteolytic cleavage of MAP3K14, thereby triggering the non-canonical NF-κB pathway ^49^. The t(11;18) is the most frequent chromosomal translocation in MALT lymphomas, detected in 15 to 50% of cases, more commonly in gastric and pulmonary MALT lymphomas ^13, 50, 51^. SMZL and NMZL present recurrent mutations of the *BIRC3* gene in about 10% and 5% of cases, respectively ^6, 31, 52^. These mutations disrupt the same RING domain that is removed by the t(11;18) in MALT lymphomas, and the mutated BIRC3 is no longer able to inactivate MAP3K14 via ubiquitination ^31, 53^. *TRAF3* is also inactivated in about 5% of SMZL and NMZL cases by mutations leading to the loss of its C-terminal MATH domain necessary for the MAP3K14 docking site and recruitment to BIRC3 degradation ^6, 31^.

The t(14;18) translocation occurs in 15 to 20% of MALT lymphomas, more frequently in non-gastrointestinal sites such as lung and ocular adnexa, and brings the intact *MALT1* gene under the control of the *IGH* enhancer, resulting in deregulated expression of *MALT1* directly contributing to NF-κB activation ^10, 54^. The t(1;14) translocation and its variant t(1;2)(p22;p12) occur in 1 to 2% of MALT lymphomas ^55^. Similarly to the t(14;18), the entire coding region of *BCL10* is moved under the control of the *IGH* enhancer region (or the *IGL*k region in the case of a variant translocation) and has a direct effect on the NF-κB signaling ^56^.

The important role of BIRC3-MALT1 fusion protein, as well as MALT1 and BCL10 upregulation, in MALT lymphoma is further underlined by mouse models with development of MALT lymphomas and DLBCL in *MALT1* gene transgenic mice ^57^, expansion of marginal zone cells in *BIRC3*- *MALT1* ^18, 35, ^and BCL10 transgenic mice ^58^.

In SMZL and NMZL, NF-κB signaling is also sustained by mutations occurring in genes coding members of upstream pathways, such as Toll-like receptor (TLR) and BCR signaling. MYD88 is an adaptor protein necessary for propagating the TLR downstream signal. MYD88 has a modular structure with an N-terminus death domain (DD), an intermediate linker domain (ID), and a C-terminus TIR domain. The DD allows the creation of a multimeric complex, via the oligomerization and interaction with the serine-threonine kinases IRAK1–4, which activate the NF-κB. *MYD88* is affected by somatic mutations in 15% of SMZLs and 10% of NMZLs and MALT lymphomas. *MYD88* mutations affect a conserved beta-beta loop of the protein TIR domain ^26, 33, 59– 69^ and lead to spontaneous and uncontrolled MYD88/IRAK complex formation ^59^. Components of the BCR pathway are also mutated, but almost exclusively in the SMZL and NMZL ^6, 25– 27, 32, 33, 52, 70^, including *CARD11*, which links the BCR to NF-κB, in 5 to 10% of cases ^6, 25– 27, 31– 33, 52, 60, 70^. CARD11 mutations cause spontaneous protein multimerization and association with CBM complex components (for example, BCL10) with IKKβ kinase-mediated NF-κB activation ^71^.

## NOTCH signaling

Similarly to NF-κB signaling, NOTCH activation is important for marginal zone differentiation and homing of B cells to the splenic marginal zone ^72– 74^. The *NOTCH2* gene is mutated in 10 to 25% of SMZLs, in about 25% of NMZLs, and in less than 5% of MALT lymphomas ^6, 32, 33, 52, 68, 75, 76^. *NOTCH1* is also mutated in about 5% of SMZLs but not at all or at a much lower frequency in NMZL and MALT lymphomas ^6, 77^. Negative regulators of NOTCH signaling (such as *SPEN*, *DTX1*, and *MAML2*) are also mutated, though at lower frequency, bringing NOTCH activation by genetic events to 40% of SMZLs and NMZLs ^6, 32, 52^. NOTCH2 and NOTCH1 are heterodimeric transmembrane proteins that, after binding with their ligands, undergo a cleavage of their intracellular portions, which, once in the nucleus, regulate gene expression via binding with transcriptional co-factors ^78, 79^. Importantly, *NOTCH1* and *NOTCH2* mutations cluster in the C-terminal PEST domain and cause a protein truncation with loss of the region necessary for inactivation via proteasomal degradation. Thus, mutations are believed to enhance the stability of the active NOTCH intracellular domains (NICDs) once it has been triggered by microenvironmental interactions ^80^.

## KLF2

Inactivating mutations in the *KLF2* gene are very frequent in SMZL (20–40%) and NMZL (20% of cases) ^52, 81, 82^. *KLF2* is a transcription factor, and mice with a B cell–specific deletion of KLF2 have an increased number of splenic marginal zone B cells ^83^. In lymphoma cells, mutated KLF2 delocalizes from the nucleus into the cytoplasm and is not able to inhibit the NF-κB signaling activated by upstream pathways, including the BCR and TLR pathways ^82^.

## PTPRD

PTPRD is a receptor-type protein tyrosine phosphatase expressed in normal germinal center B cells and, at lower levels, in marginal zone B cells ^52^. Almost exclusively in NMZL, *PTPRD* is inactivated by mutations or deletions in about 20% of cases ^52^. *PTPRD* regulates many biologic pathways, and NMZL cases with mutated PTPRD appear to have an increased cell proliferation, indicating an involvement of PTPRD in cell proliferation ^52^.

## Chromatin remodeling and epigenome regulation

As a whole, mutations in genes coding for epigenetic regulators are found in about 40% of MZLs. Although their precise consequences in MZL cells are still unknown, mutations in genes such as *KMT2D* (MLL2), *SIN3A*, *ARID1A*, *EP300*, C *REBBP*, and *TBL1XR1* highlight a deregulation of the epigenome in all three MZLs ^6, 26, 33, 52, 68, 76, 84^. The importance of epigenetic changes is also underlined by methylation changes described in SMZL, which associate with silencing of different tumor suppressor genes and over-expression of genes involved in BCR/PI3K/AKT/NF-κB signaling, PRC2-complex ( *EZH2*, *EED*, and *SUZ12*), and *MYC* and *IRF4* targets. Clinically, epigenetic changes in SMZL associate with inferior outcome and risk of transformation to a diffuse large B-cell lymphoma (DLBCL) ^85^. In MALT lymphomas, promoter methylation seems to increase with a continuum from MALT lymphoma, to MALT lymphoma with large cell component, to DLBCL. Consistently, a series of tumor suppressor genes such as *CDKN2A*, *DAPK1*, *CDH1*, and *TNFAIP3* are silenced via promoter methylation in MALT lymphoma progression ^86– 88^.

## Antigen stimulation

There is a lot of evidence supporting the notion that antigen stimulation is important for the development and progression of MZLs. MALT lymphoma arises from B cells within populations of immune cells induced by a chronic inflammation taking place in extranodal sites in organs that are physiologically devoid of germinal centers. The most frequent site of MALT lymphoma is the stomach, where the disease has been very clearly associated with the chronic gastritis induced by *Helicobacter pylori* ^89^. MALT lymphomas arising in other anatomical sites have also been associated with additional infectious agents, although the etiologic link is not as strong as for the gastric localization and *H. pylori* ^89^. These include *Clamydophila psittaci* in orbital adnexa MALT lymphoma ^90– 97^, *Borrelia burgdorferi* in cutaneous MALT lymphoma ^98– 100^, *Campylobacter jejuni* in immunoproliferative small intestine disease ^101, 102^, *Achromobacter xylosoxidans* in pulmonary MALT lymphoma ^103^, and hepatitis C virus (HCV) in all MZLs ^104– 108^. Besides infection, chronic inflammations in the context of autoimmune disorders, such as Sjögren syndrome or Hashimoto’s thyroiditis, are strongly associated with the development of MALT lymphomas affecting salivary glands and thyroid, respectively ^109– 117^. Besides the continuous antigenic stimulation, oncogenic events, such as those presented above, contribute to lymphoma growth and progression up to the development of frank tumor independent of the antigenic drive ^89^.

MZLs present somatically mutated immunoglobulin heavy variable (IGHV) genes in nearly all cases with a pattern of somatic hypermutation and rearrangements indicative of an antigen selection ^118– 125^. The presence of the so-called ongoing mutations (intraclonal variation) and the biased usage of some IGHV segments indicate that the expansion of lymphoma cells could still be antigen-driven. In MALT lymphomas, there is an apparently biased usage of different IGHV families in cases derived from different anatomical sites or with particular clinical and genetic features: IGHVH1-69 in salivary gland lymphomas, IGHVH3-30 or IGHVH3-23 in gastric MALT lymphomas responsive to *H. pylori* eradication and without the t(11;18) translocation, IGHVH4-34 in orbital adnexal lymphomas, IGHV3 and IGHV4 families in pulmonary lymphomas, and IGHVH1-69 or IGHVH4-59 in cutaneous lymphomas ^126^. Similarly, a biased IGHV usage is present in SMZL with a stereotyped BCR in about 10% of cases ^127^ and a biased usage of the IGHV 1-2*04 allele in about 30% of cases ^123, 127– 130^ and in NMZL with a biased usage of the *IGHV4-34* gene in 20 to 30% of cases ^131, 132^. IGHVH1-69 is also frequently detected in HCV-related MZLs, similarly to what observed in other HCV-related B-cell expansions such as in the monoclonal rheumatoid factor-like IgM component of the type II mixed cryoglobulinemia, and in monoclonal paraproteins from patients with HCV infection ^106– 108, 133^. Finally, the antibodies expressed by MALT lymphoma and SMZL cells often recognize self-antigens ^134– 136^.

## Clinical implications

Molecular lesions may be of help to inform MZL diagnosis, prognosis, and therapeutic targeting. In general, the presence of trisomies of 3 and 18 as single lesions or associated only with TNFAIP3 loss or 7q deletions is highly indicative of MZL more than other small cell lymphomas. The presence of translocations affecting *MALT1* and *BIRC3* is basically exclusive to MALT lymphoma, in which they are associated with lower response rate to antibiotics treatment. From a diagnostic standpoint, *NOTCH2* mutations are highly specific for SMZL and NMZL among mature B-cell tumors, including conditions that look alike, thus representing a biomarker with positive predictive value for non-MALT MZL specification. Within non-MALT MZL, *PTPRD* mutations are enriched in NMZL and thus may represent a genetic biomarker that, though not highly sensitive, is provided with a positive predictive value for NMZL specification.

From a prognostic standpoint, *KLF2* mutations and *NOTCH2* mutations represent promising prognostic biomarkers associated with poor survival and transformation to aggressive lymphoma whose broad application in clinical practice requires the assessment of whether their incorporation into the currently available clinical prognostic models improves risk stratification of patients.

Molecular aspects of MZL point to deregulated cellular programs worth exploring as therapeutic targets. Pharmacologic interference of NOTCH signaling, non-canonical NF-κB signaling, or upstream pathways that are connected to NF-κB, including BCR signaling, are attractive approaches in these lymphomas.
